# The Efficacy and Safety of 12 Weeks of Sofosbuvir and Ledipasvir versus Sofosbuvir, Ledipasvir, and Ribavirin in Patients with Chronic Hepatitis C, Genotype 1, Who Have Cirrhosis and Have Failed Prior Therapy: A Systematic Review and Meta-Analysis

**DOI:** 10.1155/2017/6468309

**Published:** 2017-03-06

**Authors:** William Stokes, Carol Fenton, Fiona Clement, Matthew James, Paul Ronksley, Karen L. Tang

**Affiliations:** ^1^Department of Medicine, University of Calgary, Foothills Medical Centre, 1403 29th St. NW, Calgary, AB, Canada T2N 2T9; ^2^Department of Community Health Sciences, University of Calgary, Teaching, Research and Wellness Building, 3280 Hospital Drive NW, Calgary, AB, Canada T2N 4N1

## Abstract

*Background.* The recommended therapy for patients with chronic hepatitis C (CHC), genotype 1, who have cirrhosis and have failed prior therapy is 12 weeks of sofosbuvir (SOF), ledipasvir (LDV), and ribavirin (RBV). This recommendation is based on expert opinion, and the efficacy of 12 weeks of SOF/LDV compared to SOF/LDV/RBV in this patient population has not yet been established.* Methods*. We conducted a systematic review and meta-analysis. Two investigators independently searched electronic databases and relevant conference proceedings for randomized controlled trials comparing rates of sustained virologic response 12 weeks after therapy (SVR12) when using 12 weeks of SOF/LDV versus 12 weeks of SOF/LDV/RBV in patients with CHC, genotype 1, who have cirrhosis and failed previous therapy.* Results.* Our search strategy yielded 596 studies of which four met criteria for inclusion. The pooled RR of not achieving SVR12 with SOF/LDV versus SOF/LDV/RBV was 1.21 (95% CI: 0.42–3.48). Adverse events were lower in the SOF/LDV compared to the SOF/LDV/RBV arms (pooled RR: 0.11, 95% CI: 0.04–0.29).* Conclusions.* Our findings suggest that 12 weeks of SOF/LDV cannot be considered noninferior to 12 weeks of SOF/LDV/RBV to achieve SVR12 in patients with CHC who have cirrhosis and failed prior therapy.

## 1. Introduction

Over 210,000 Canadians are estimated to be currently living with chronic hepatitis C (CHC) infections [1]. To date, liver failure due to CHC is still the number one indication for liver transplant in Canada [2]. The number of Canadians with advanced CHC that develop cirrhosis is increasing annually and is expected to peak between 2031 and 2035 at approximately 40,000 per year [3]. Hepatitis C-related healthcare costs are forecasted to increase by 60% between 2013 and 2032, mostly due to cirrhosis and associated complications [3].

In Canada, 12 weeks of sofosbuvir and ledipasvir is the first-line therapy for patients without comorbidities with CHC, genotype 1, who are treatment naïve. However, certain characteristics put patients at higher risk of treatment failures. This includes patients who have previously failed therapy, or “null responders,” and those who have cirrhosis. For both patients with cirrhosis and those who have failed previous therapy, current guidelines recommend the use of either 12 weeks of SOF/LDV/RBV or 24 weeks of SOF/LDV alone [4]. These guidelines are based only on expert opinion, as there was limited evidence available on the effectiveness of the new medications in these populations.

In the ION2 study, 12 weeks of SOF/LDV, 12 weeks of SOF/LDV/RBV, and 24 weeks of SOF/LDV were compared to each other for patients with hepatitis C, genotype 1, who have cirrhosis and failed prior therapy [5]. In this subgroup of patients, SVR12 was found to be 99% with 24 weeks of LDV/SOF, 86% with 12 weeks of LDV/SOF, and 82% with 12 weeks of LDV/SOF/RBV. The study therefore suggested that 24 weeks of SOF/LDV is better than 12 weeks of SOF/LDV or 12 weeks of SOF/LDV/RBV in patients with CHC, genotype 1, who have cirrhosis and failed prior therapy. However, the subsequent SIRIUS study refuted ION2's results by demonstrating similarly high rates of SVR12 between 24 weeks of SOF/LDV (97%) and 12 weeks of SOF/LDV/RBV (96%) [6]. As a result, current guidelines recommend either 24 weeks of SOF/LDV or 12 weeks of SOF/LDV/RBV for patients with CHC, genotype 1, who have cirrhosis and failed prior therapy [7]. Unfortunately, the SIRIUS trial did not include 12 weeks of SOF/LDV. Therefore, it is unclear whether 12 weeks of SOF/LDV/RBV is superior to 12 weeks of SOF/LDV. Other studies comparing 12 weeks of SOF/LDV versus SOF/LDV/RBV in patients with hepatitis C, genotype 1, who have cirrhosis and failed prior therapy show results that suggest that 12 weeks of SOF/LDV versus SOF/LDV/RBV in this population may have similar results, but no pooled systematic review and meta-analysis has been done to determine whether any differences among the two treatments exist [5, 8–10].

We undertook a systematic review and meta-analysis of randomized controlled trials to determine the efficacy of 12-week therapy with SOF/LDV compared to SOF/LDV/RBV in achieving SVR12 in cirrhotic patients with chronic hepatitis C, genotype 1, who have previously failed therapy. Our secondary objective was to determine the risk of adverse events for both treatment regimens.

## 2. Methods

### 2.1. Search Strategy

We performed a systematic review and meta-analysis using a predetermined protocol and in accordance with PRISMA standardized reporting guidelines [11]. Two investigators (WS and CF) independently searched electronic databases from inception to November 8, 2015, including PubMed, the Central Registry of Controlled Trials of the Cochrane Library, MEDLINE, EMBASE, clinicaltrials.org, and Science Citation Index Expanded. No language restrictions were applied. Our search strategy combined the names and alternate names of sofosbuvir and ledipasvir using the Boolean operator AND to map (search by keyword) and explode (search by subject heading where appropriate). Alternative names of sofosbuvir and ledipasvir, or their active metabolites, were combined using the Boolean operator OR. Harvoni®, the combination brand name for ledipasvir/sofosbuvir, was also searched and combined with the above terms using the Boolean operator OR (Supplemental Appendix  A, available online at https://doi.org/10.1155/2017/6468309). Our search was broad in order to capture all relevant publications given the relative novelty of sofosbuvir and ledipasvir combination therapy.

Conference proceedings from the American College of Gastroenterology Annual Scientific Meetings, Annual Meetings of the American Association for the Study of Liver Diseases, and Annual Meetings of the Infectious Diseases Society of America were also searched for relevant abstracts. Conference proceedings were searched from 2010 onwards as ledipasvir was not chemically identified until 2013 [12]. Finally, experts in the field and Gilead Sciences were contacted for other published work and reference lists of all relevant publications were manually searched.

### 2.2. Identification of Articles for Systematic Review and Meta-Analysis

All identified abstracts were screened for full-text review by two investigators (WS and CF). Interrater agreement was measured using the kappa statistic. Disagreements were resolved by consensus. Articles were included if they were randomized clinical trials presenting original data comparing the combinations of 12 weeks of SOF/LDV and SOF/LDV/RBV in individuals with chronic hepatitis C, genotype 1. We analyzed genotypes 1a and 1b together since the two could not be independently assessed for patients with cirrhosis who failed previous therapy without access to individual patient data. Trials that did not have study arms of cirrhotic patients who failed previous therapy were excluded. Trials that did not measure sustained virologic response at 12 weeks after treatment were also excluded.

### 2.3. Data Extraction and Quality Assessment

Data extraction was performed independently by the same two investigators (WS and CF). Data extracted included study characteristics (sites involved, year, location, and inclusion/exclusion criteria), patient characteristics (genotype 1 subtype, age, sex, percentage of patients who failed previous therapy, percentage of patients with cirrhosis, and percentage of patients with non-CC interleukin 28B gene locus (IL28B) genotypes), treatment characteristics (doses and duration), and outcome characteristics (number of SVR12, dropouts, viral breakthrough, viral relapse, and adverse events). Study quality was also independently assessed using the Cochrane Collaboration tool for assessing risk of bias [13]. The Cochrane Collaboration elements for assessing risk of bias include random sequence generation, allocation concealment, blinding, attrition bias, reporting bias, and other biases (such as conflicts of interest through industry funding).

### 2.4. Data Synthesis and Analysis

Failure to achieve SVR12 among cirrhotic patients who failed previous CHC therapy with IFN based or protease inhibitor based regimens was assessed using relative risk (RR), with the reference group being patients receiving 12 weeks of LDV/SOF/RBV. Study subjects lost to follow-up were included as virologic failures. Pooled estimates were derived using DerSimonian and Laird random effects models given the observed differences in patient characteristics between studies. Pooled RRs were visually demonstrated using forest plots and heterogeneity among studies was assessed using the *I*^2^ and Cochran *Q* statistics. The relative risks of adverse effects of 12 weeks of SOF/LDV versus SOF/LDV/RBV were also pooled using random effects models, with patients receiving 12 weeks of LDV/SOF/RBV as the reference group. As a sensitivity analysis for all pooled estimates, fixed effect models were generated to assess the robustness of the findings. Finally, small study effects, potentially indicative of publication bias, were assessed visually through a funnel plot and tested for using Begg and Mazumdar's rank correlation test for asymmetry [14]. A significant statistical test (*p* < 0.05) or observed funnel plot asymmetry suggests small study effects that may potentially be caused by publication bias. All analyses were conducted using STATA 14.0 (StataCorp., College Station, TX, USA).

## 3. Results

### 3.1. Search Results

**Figure 1:**
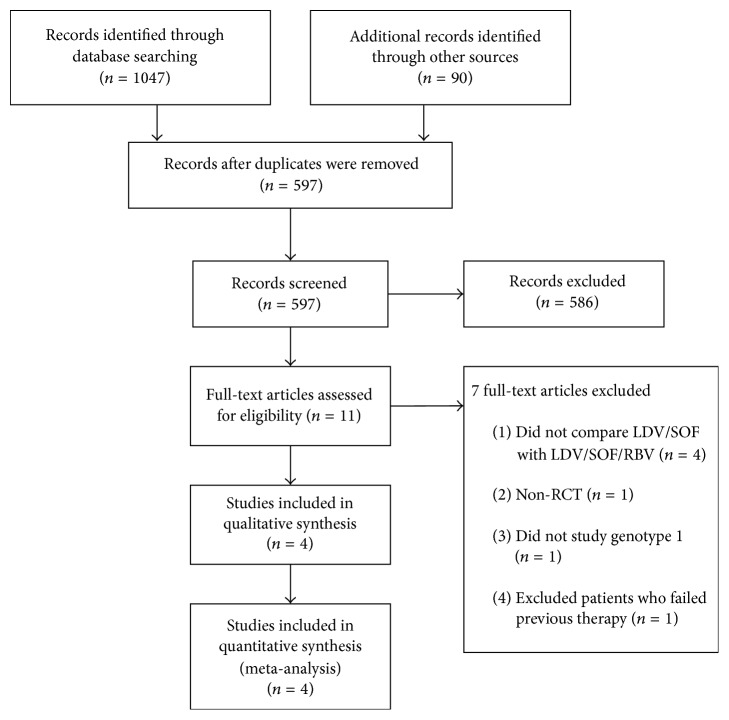
Search strategy results using PRISMA flow diagram [26].

Our search strategy yielded 597 abstracts, of which 586 were excluded for not meeting inclusion/exclusion criteria (Figure 1). Subsequently, 11 papers were included for full-text review, of which 4 papers met inclusion criteria for inclusion in the systematic review [5, 8–10]. Agreement was excellent between the two investigators (kappa statistic = 1.0 for the 4 included papers).

### 3.2. Study Characteristics

Characteristics of previously treated subjects from the 4 included studies are provided in Table 1. Two studies were phase II trials and two were phase III. Studies varied widely in terms of geographic location, hepatitis C genotype 1 subtypes, and patient characteristics. Studies were carried out in USA, New Zealand, or Japan. Study participants were, on average, middle aged. Men represented the majority of participants in all studies except for one [10]. All studies included participants with cirrhosis, but in frequencies among the previously treated subgroups ranging from 20% to 100%. Furthermore, all studies had different proportions of subgenotype 1a or 1b included, ranging from 13% to over 96% genotype 1a. Among all studies, patients with significant comorbidities including human immunodeficiency virus, hepatitis B, and decompensated cirrhosis were excluded.

All trials used the same dose of SOF/LDV/RBV except for Mizokami et al., who used a lower RBV dose as recommended by the manufacturers for Japanese patients. Outcome measurements were consistent across studies and were defined as undetectable HCV after 12 weeks of therapy using COBAS TaqMan HCV test for HCV RNA detection. Undetectable HCV was defined as <25 IU/mL for all studies except for the ELECTRON trial in which undetectability was defined as <43 IU/mL. All studies had fewer than 2% of subjects lost to follow-up [9].

Cochrane Risk of Bias Assessment yielded similar risks for all included studies (Table 2). Studies were generally high in quality, except for the presence of a significant risk of bias associated with nonblinding and for being funded and conducted by the makers of sofosbuvir and ledipasvir (Gilead Sciences).

### 3.3. Sustained Virologic Response

Overall, failure to achieve SVR12 occurred in 0–30% and 0–19% of cirrhotic patients who failed previous therapy who underwent 12 weeks of therapy with SOF/LDV and SOF/LDV/RBV, respectively. Of note, the ELECTRON trial was an outlier among study results, having 30% SVR12 failure rates in the LDV/SOF group compared to the 0% to 14% among the other studies [9].

**Figure 2:**
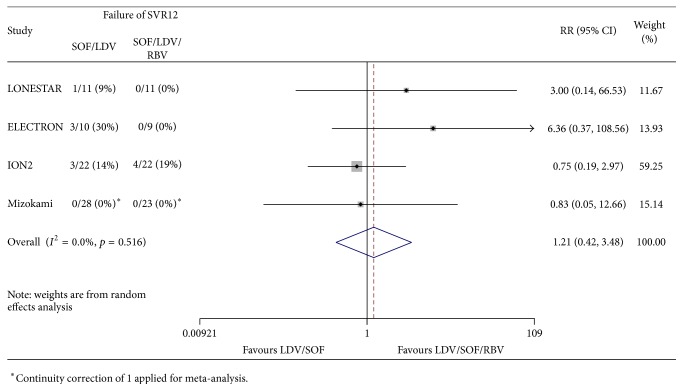
Forest plot of relative risks of not achieving SVR12 with 12 weeks of SOF/LDV versus SOF/LDV/RBV in chronic hepatitis C, genotype 1, of cirrhotic patients who have failed previous therapy.

Among cirrhotic patients who failed previous therapy, the pooled RR of not achieving SVR12 after completing 12 weeks of SOF/LDV therapy compared to 12 weeks of SOF/LDV/RBV was 1.21 (95% CI: 0.42–3.48) (Figure 2). Unfortunately, SVR for genotype subtype 1a could not be adequately assessed among studies without access to individual patient data. To determine whether genotype subtype had a significant impact among cirrhotic patients who failed previous therapy, we performed sensitivity analysis by excluding Mizokami et al.'s study, as 96% of its study participants were genotype 1b compared to 13–21% among the other studies [10]. Excluding Mizokami et al.'s study yielded minimal changes, with the pooled RR of not achieving SVR12 increasing slightly to 1.39 (95% CI: 0.39–4.97). Though with limited capacity of detecting heterogeneity with low number of studies, in the overall results, the Cochran *Q* test statistic was 0.516 indicating homogeneity among studies. *I*^2^, despite being also limited by small sample size, was 0%. Fixed effect modeling did not change the statistical significance of any result.

### 3.4. Adverse Events

We were unable to assess adverse events in cirrhotic patients only, so we have presented the pooled overall results for these outcomes. Adverse events were significantly more common in patients who received SOF/LDV/RBV compared to those who received SOF/LDV. The pooled relative risk for having any adverse event in LDV/SOF compared to LDV/SOF/RBV was 0.11 (95% CI: 0.04–0.29) (Table 3). Differences in adverse events were observed for those known to be associated with ribavirin, including fatigue/asthenia, rash, irritability, cough/bronchitis, and anemia. Three patients in the SOF/LDV/RBV arms discontinued study drugs due to adverse events. These adverse events included cardiac arrest, ribavirin-associated morbilliform drug eruption, and peritonitis secondary to perforated diverticulum, of which the two former events were felt to be related to study treatment. Serious adverse events were higher in SOF/LDV/RBV versus the SOF/LDV arms but differences were not statistically significant. A total of four serious adverse events were thought to be related to study therapy and all occurred in the SOF/LDV/RBV arms. These were hemolytic anemia, nonfatal myocardial infarction, and the aforementioned morbilliform rash and cardiac arrest.

### 3.5. Publication Bias

**Figure 3:**
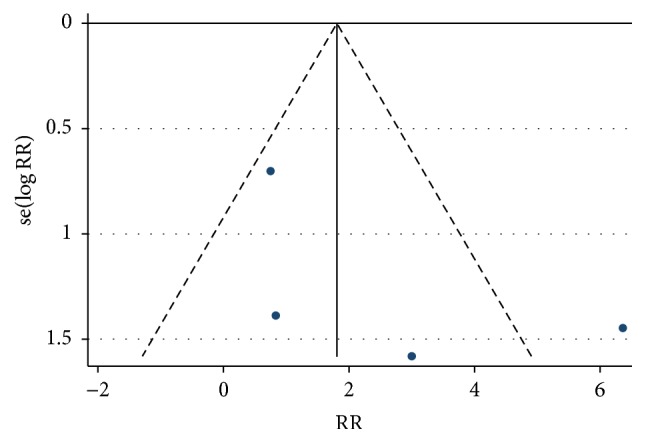
Assessment of publication bias using funnel plot with pseudo 95% confidence limits.

Although assessment for publication bias was conducted, interpretation of results was significantly limited by the small number of studies involved. The associated funnel plot shows visual asymmetry, suggesting that publication bias may be present (Figure 3). The funnel plot also demonstrates an outlier, which corresponds to the ELECTRON trial [9].

## 4. Discussion

The results of our systematic review and meta-analysis suggest that 12 weeks of SOF/LDV cannot be considered noninferior to 12 weeks of SOF/LDV/RBV in achieving SVR12 in patients with hepatitis C, genotype 1, who have cirrhosis and have previously failed therapy. When examining the pooled point estimate, the pooled effect size is in keeping with over a 20% increase in risk of failure to achieve remission. Also, the range of plausible effect illustrates the imprecision of the pooled estimate, with as much as a 3-fold potential increase in risk based on the pooled effect seen from the existing studies, beyond an acceptable threshold for SOF/LDV to be considered noninferior. Our results therefore support continued adherence to recent guidelines, which recommend the preferential use of 12-week SOF/LDV/RBV over 12-week SOF/LDV alone in this subgroup until more research has been conducted [4].

Results from our systematic review indicate that RCTs examining 12 weeks of LDV/SOF versus LDV/SOF/RBV in patients with hepatitis C, genotype 1, who have cirrhosis and previously failed therapy are of moderate quality. All studies were nonblinded, which may be due to requirements to monitor adverse effect profiles, including anemia. However, there is concern regarding pharmaceutical company Gilead Sciences' substantial role in funding and coordinating the studies. While steps are often taken to minimize bias in company sponsored trials, it is known that these trials tend to favour the company's product compared to independently conducted trials regardless of trial methodology [15–17].

As expected, adverse events were higher among SOF/LDV/RBV compared to SOF/LDV and were due to adverse events that are typically associated with ribavirin treatment such as anemia, fatigue, rash, and cough. Serious events, including those associated with discontinuation of study drug, were more likely to occur with SOF/LDV/RBV treatment but the difference was not statistically significant between groups. Use of SOF/LDV/RBV over SOF/LDV may further increase risk of nonadherence by increasing pill burden and pill frequency [18]. Determining noninferiority between SOF/LDV/RBV and SOF/LDV is therefore an important avenue to continue researching since eliminating RBV from treatment regimens has potential to improve adherence rates in real world settings.

Our study is the first systematic review and meta-analysis of randomized controlled trials comparing 12 weeks of SOF/LDV versus 12 weeks of SOF/LDV/RBV in patients with hepatitis C, genotype 1, who have cirrhosis and have failed previous therapy. Our study does have several limitations, however. We were unable to prove noninferiority given our very wide confidence intervals in the pooled effect estimates. Estimating 95% success rates for LDV/SOF and LDV/SOF/RBV and using a noninferiority limit of 5%, a clinical trial with sample size of 652 patients (326 per arm) would be needed to prove noninferiority with 90% power and significance level of 5% [19]. Moreover, trials included in our study differed widely. Although we were able to extract data for cirrhotic patients who failed previous therapy, subgroup analysis (such as for the subgroup with HCV genotype subtype 1a) was limited without having individual patient data. Individual patient data is important in the context of HCV, where clinical heterogeneity that could influence the outcome of SVR may not be adequately documented at the aggregate level. Therefore, more studies, especially those independent of pharmaceutical involvement, are needed to more accurately compare the degree of efficacy between LDV/SOF and LDV/SOF/RBV.

Although newer HCV treatment regimens have emerged, SOF/LDV continues to be relevant in the treatment for HCV, genotype 1, in patients with cirrhosis who previously failed therapy. Elbasvir/grazoprevir/ribavirin is effective for patients with HCV, genotype 1, who previously failed treatment but requires frequent monitoring in patients with cirrhosis [20]. Sofosbuvir/daclatasvir/ribavirin can be used in cirrhotic patients who previously failed therapy but require longer treatment durations (24 weeks) [21]. The most promising newest HCV treatment regimen is sofosbuvir/velpatasvir (SOF/VEL), which was approved by Health Canada in July 2016 for the treatment of all genotypes of hepatitis C, including patients with cirrhosis who failed previous therapy [22]. Similar to SOF/LDV, SOF/VEL offers once daily dosing for a total of 12 weeks and has a largely benign side effect profile. SOF/VEL has the added benefit of not requiring ribavirin for treatment of genotype 1 cirrhotic patients who failed previous therapy and in fact may be cheaper, though the direct patient cost is not yet fully known [23]. However, SOF/LDV still has several advantages of SOF/VEL in Canada. Currently, SOF/VEL is not yet widely prescribed in Canada since SOF/VEL is not covered by most provincial health systems while SOF/LDV is. In addition, cheaper generic versions of SOF/LDV will become available before SOF/VEL since SOF/LDV's patent was approved in 2014 [24], compared to 2016 [25] for SOF/VEL. Therefore, even in the context of new and emerging HCV therapies, SOF/LDV and SOF/LDV/RBC remain important for cirrhotic patients who failed prior therapy. An understanding of the optimal treatment duration and regimen for these two treatment options continues to be crucial.

In conclusion, our study could not determine whether 12 weeks of SOF/LDV is noninferior to 12 weeks of SOF/LDV/RBV for achieving SVR12 in cirrhotic patients who failed previous therapy with chronic hepatitis C, genotype 1. Determining whether 12 weeks of LDV/SOF for cirrhotic patients who have failed previous therapy is as effective as 12 weeks of SOF/LDV/RBV, or the alternatively approved regimen of 24 weeks of SOF/LDV, is an important avenue to continue exploring. Using 12 weeks of SOF/LDV over 24 weeks of LDV/SOF has numerous benefits including substantial cost saving; the shorter duration of therapy may also be critical in patients who are intolerant or ineligible for ribavirin treatment or in whom ribavirin may not be favoured, such as patients at high risk for nonadherence. Further high-quality randomized controlled trials are required to assess the optimal drug regimen and duration in cirrhotic patients with chronic hepatitis C, genotype 1, who have previously failed therapy.

## Supplementary Material

A draft of our MEDLINE search strategy is available in Appendix A.

## Figures and Tables

**Table 1:** Study characteristics of the previously treated subgroups within included studies.

Study	LONESTAR [8]	ELECTRON [9]	ION2 [5]	Mizokami et al. [10]
Publication year	2014	2014	2014	2015
Sample size of previously treated subjects given 12 weeks of therapy	40	19	220	175
Cirrhosis	55%	100%	20%	29%
Geographical location	USA	New Zealand	USA	Japan
Funding source	Industry	Industry	Industry	Industry
Number lost to follow-up	0	0	0	0
Average age (years)	53	59	57	35% > age 65
Male gender	73%	95%	66%	43%
Genotype 1a	86%	81%	79%	4%
Genotype 1b	14%	19%	21%	96%
Black race	10%	0%	18%	0%
Mean BMI (Kg/m^2^)	31.5	29.2	29	62% < 25 Kg/m^2^
Mean HCV RNA (log_10_ IU/mL)	6.3	6.4	6.5	87% > 800,000 IU/mL

**Table 2:** Cochrane Risk of Bias Assessment for included studies.

	LONESTAR	ELECTRON	ION2	Mizokami
Random sequence generation	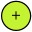	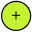	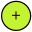	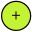
Allocation concealment	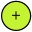	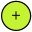	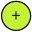	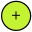
Blinding	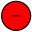	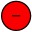	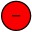	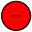
Attrition bias/incomplete outcome data	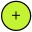	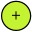	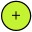	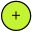
Reporting bias/selective reporting	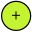	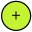	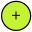	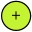
Other biases (funding)	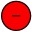	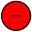	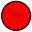	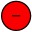

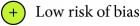

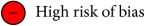

**Table 3:** Combined adverse events for SOF/LDV and SOF/LDV/RBV and their associated pooled relative risks, with SOF/LDV/RBV as the reference group.

Adverse effect	SOF/LDV (*N* = 328)	SOF/LDV/RBV (*N* = 344)	Pooled RR (95% CI)
Any side effect	223	523	0.11 (0.04–0.29)
Discontinuation because of adverse effect	0	3	0.66 (0.11–3.84)
Serious	5	7	1.09 (0.64–1.85)
Fatigue/asthenia	33	68	0.57 (0.39–0.83)
Rash	2	18	0.20 (0.06–0.66)
Irritability	2	18	0.19 (0.05–0.70)
Cough/bronchitis	7	23	0.39 (0.17–0.88)
Anemia	3	45	0.11 (0.04–0.24)
HA	44	61	0.97 (0.77–1.24)
Insomnia	10	27	0.67 (0.40–1.10)
Nausea	19	43	0.73 (0.51–1.05)
Diarrhea	7	8	1.16 (0.71–1.90)
URTI	7	23	0.50 (0.21–1.19)
Muscle spasm	1	10	0.29 (0.08–1.06)
Arthralgia	7	20	0.66 (0.36–1.20)
Dry skin	0	10	0.26 (0.04–1.73)
Dizziness	3	13	0.52 (0.21–1.30)
Dyspnea	0	21	0.11 (0.01–1.59)
Hgb < 8.5 g/dL	1	9	0.58 (0.07–4.750)
Lymphocytes < 350 per mm^3^	0	1	N/A^*∗*^
Neutrophils 500–750 per mm^3^	2	0	N/A^*∗*^
Platelets 25–50 per mm^3^	2	0	1.51 (0.85–2.68)

^*∗*^Pooled result not possible as adverse event recorded in one study only.
