# Supplementary material for: The Efficacy and Safety of 12 Weeks of Sofosbuvir and Ledipasvir versus Sofosbuvir, Ledipasvir, and Ribavirin in Patients with Chronic Hepatitis C, Genotype 1, Who Have Cirrhosis and Have Failed Prior Therapy: A Systematic Review and Meta-Analysis
Source: Can J Gastroenterol Hepatol. 2017 Mar 6;2017:6468309. doi: 10.1155/2017/6468309 (PMC5358456; doi:10.1155/2017/6468309)
Supplement: Supplementary file 1 — A draft of our MEDLINE search strategy is available in Appendix A. [file 6468309.f1.docx]

**APPENDIX A: Search Strategy (Ovid MEDLINE)**

Database: Ovid MEDLINE

Search Strategy:

1. Harvoni.mp
2. exp sofosbuvir or sofosbuvir.mp
3. Sovaldi.mp or Hepcinat.mp or Hepcvir.mp or Resof.mp or SoviHep.mp or Virunon.mp or GS 7977.mp or GS-7977.mp or GS7977.mp or GS 331007.mp or GS-331007.mp or GS331007.mp or GS 461203.mp or GS-461203.mp or GS461203.mp or PSI 7977.mp or PSI-7977.mp or PSI7977.mp or SOF.mp
4. Ledipasvir.mp or GS-5885.mp or GS 5885.mp or GS5885.mp or WHO9796.mp or WHO 9796.mp or WHO-9796.mp or or LDV.mp
5. #2 or #3
6. #5 and #4
7. #1 or #6
